# *In Vivo* Recording of Neural and Behavioral Correlates of Anesthesia Induction, Reversal, and Euthanasia in Cephalopod Molluscs

**DOI:** 10.3389/fphys.2018.00109

**Published:** 2018-02-20

**Authors:** Hanna M. Butler-Struben, Samantha M. Brophy, Nasira A. Johnson, Robyn J. Crook

**Affiliations:** Department of Biology, San Francisco State University, San Francisco, CA, United States

**Keywords:** general anesthesia, local anesthesia, analgesia, immobilization, cephalopoda, welfare impact, neurophysiology

## Abstract

Cephalopod molluscs are among the most behaviorally and neurologically complex invertebrates. As they are now included in research animal welfare regulations in many countries, humane and effective anesthesia is required during invasive procedures. However, currently there is no evidence that agents believed to act as anesthetics produce effects beyond immobility. In this study we demonstrate, for the first time, that two of the most commonly used agents in cephalopod general anesthesia, magnesium chloride and ethanol, are capable of producing strong and reversible blockade of afferent and efferent neural signal; thus they are genuine anesthetics, rather than simply sedating agents that render animals immobile but not insensible. Additionally, we demonstrate that injected magnesium chloride and lidocaine are effective local anesthetic agents. This represents a considerable advance for cephalopod welfare. Using a reversible, minimally invasive recording procedure, we measured activity in the pallial nerve of cuttlefish (*Sepia bandensis*) and octopus (*Abdopus aculeatus, Octopus bocki*), during induction and reversal for five putative general anesthetic and two local anesthetic agents. We describe the temporal relationship between loss of behavioral responses (immobility), loss of efferent neural signal (loss of “consciousness”) and loss of afferent neural signal (anesthesia) for general anesthesia, and loss of afferent signal for local anesthesia. Both ethanol and magnesium chloride were effective as bath-applied general anesthetics, causing immobility, complete loss of behavioral responsiveness and complete loss of afferent and efferent neural signal. Cold seawater, diethyl ether, and MS-222 (tricaine) were ineffective. Subcutaneous injection of either lidocaine or magnesium chloride blocked behavioral and neural responses to pinch in the injected area, and we conclude that both are effective local anesthetic agents for cephalopods. Lastly, we demonstrate that a standard euthanasia protocol—immersion in isotonic magnesium chloride followed by surgical decerebration—produced no behavioral response and no neural activity during surgical euthanasia. Based on these data, we conclude that both magnesium chloride and ethanol can function as general anesthetic agents, and lidocaine and magnesium chloride can function as local anesthetic agents for cephalopod molluscs.

## Introduction

Cephalopods are used frequently in studies of camouflage, motor control, cognition, visual processing, environmental toxicology, and microbiology (Hanlon and Messenger, 1996; Dickel et al., 2001; Sumbre et al., 2001; Darmaillacq et al., 2004; Lee et al., 2009; Mäthger et al., 2009; Allen et al., 2014; Chiao et al., 2015; Zepeda et al., 2017). In the course of such studies, it may be necessary to anesthetize, immobilize or euthanize cephalopods. Although there is no direct evidence that cephalopods experience pain or distress as a result of noxious sensory input (Crook and Walters, 2011; Crook, 2013; Crook et al., 2013; Hague et al., 2013; Alupay et al., 2014), in many nations cephalopods are included in regulations that govern the ethical use of vertebrate animals in research, and such legislation [for example, in the UK Scientific Procedures Act (1986), EU Directive 2010/63/EU, and the Canadian Council on Animal Care] requires that anesthesia be provided during potentially harmful procedures. Thus, a major compliance challenge in cephalopod research is the lack of data on the efficacy of commonly used “anesthetic” agents on sensation, perception and states of consciousness or unconsciousness.

A recent review article (Fiorito et al., 2015) compiled a list of some 48 studies in which procedures for “anesthetizing” cephalopods were described. Of these, 17 used high concentrations of magnesium chloride, 23 used some concentration of ethanol in seawater, 3 used a combination of these two agents, and 5 used a different agent. Notably, none of these studies used “modern” volatile or injectable anesthetic agents that are standard in the fields of vertebrate animal anesthesia (e.g., volatiles such as Isoflourane or Sevoflourane, or injectables such as Ketamine or Propofol). Instead, the field relies overwhelmingly on magnesium chloride and ethanol, which are generally considered effective for immobilization and from which cephalopods recover reliably.

A striking feature of all these studies is the lack of evidence for anesthetic efficacy that goes beyond immobility; all the measures of “anesthesia” depth reported in previous studies are behavioral indicators that cannot discriminate between paralysis and lack of sensation or loss of consciousness. Typically, anesthesia is expected to achieve four main effects: to block movement in response to stimulation (immobility), to block noxious sensation and pain (anti-nociception and analgesia) to render the subject “unrousable” (unconsciousness), and to prevent memory of the event (anteriograde amnesia; Villars et al., 2004). To date, there is no evidence that either magnesium chloride or ethanol have effects on three of these four components of anesthesia. Neural recordings of the response of the cephalopod nervous system to these two agents have never been attempted, and as such, there is currently no empirical support for the belief that their use during invasive and potentially painful procedures enhances animal welfare.

Effects of anesthetic substances have been examined in some detail in isolated neuronal cells of other molluscs (Winlow et al., 1991, 1995; Yar et al., 1993). In some gastropods and the sea-slug Aplysia, large, identified sensory and motor neurons can be co-cultured and synapses between the two can be formed *in vitro* (Carew et al., 1981; Kandel and Schwartz, 1982; Spencer et al., 1995; Winlow et al., 1995), allowing for detailed mechanistic studies of effects of various anesthetics on sensory neurons, motor neurons and their synapses (Girdlestone et al., 1989; Qazzaz and Winlow, 1999; Onizuka et al., 2005). Cephalopod neurons are not amenable to such studies; somata of most neurons are small (<10 um) (Novicki et al., 1990; Wollesen et al., 2010; Bellier et al., 2017), and isolated cells do not form synapses in culture. There are no identified sensory neurons in the cephalopod nervous system (and very limited numbers of identified motor neurons; Saidel and Monsell, 1986). The location of sensory neuron cell bodies in cephalopods is unknown, and the circuits that underlie sensation and perception remain completely undescribed (Crook and Walters, 2011; Crook, 2013). Although there have been a number of studies of synaptic mechanisms in slice preparations of the octopus brain, only one or two identified synapses have been studied, and both are interneuron-interneuron (Hochner et al., 2003; Shomrat et al., 2008; Hochner and Shomrat, 2013).

Despite these considerable limitations, laboratories in regulated countries that use cephalopods in research are charged with providing “safe, effective, and humane” anesthesia and euthanasia (Andrews et al., 2013; Smith et al., 2013; Fiorito et al., 2015). The difficulties associated with measuring sensation at the behavioral level in an immobilized animal, the lack of information about neural effects, and the field-standard use of putative anesthetic substances that are not typically in use in any other taxon, have resulted in the imposition of a regulatory requirement that is currently impossible for researchers to satisfy; without evidence that these field-standard methods produce desirable effects of anesthesia beyond immobility, it is impossible to conclude that the animal's welfare is being protected.

In this study, we aimed to investigate the effect of the two most commonly used “anesthetic” substances in cephalopod research, (magnesium chloride and ethanol) on afferent and efferent neural activity in cephalopods. The explicit goal of this work is to provide cephalopod researchers with empirical support for the assumption that these two agents protect animal welfare. Secondarily, we aim to provide a set of behavioral criteria that reliably correlate with loss of afferent neural signal (loss of sensation) and loss of efferent signal (loss of consciousness), such that researchers who lack equipment to monitor neural effects can make reasonable assumptions about anesthesia efficacy based on behavioral indicators. We do not suggest that cephalopods are potential models for mechanistic anesthesia studies, nor do we rule out that other, more modern drugs may be equally or more effective at achieving anesthesia in cephalopods. Instead, we present the first strong evidence that bath applications of either magnesium chloride or ethanol are effective at blocking afferent signal to the CNS, blocking efferent signal from the CNS, and blocking reflex and voluntary movements. In addition, we demonstrate that local anesthesia using either injected lidocaine or high concentrations of magnesium chloride is effective at blocking afferent signal and behavioral responses to noxious stimulation over the injected area;

We demonstrate that these agents, although not commonly accepted as anesthetics in other taxa (with the exception of lidocaine), achieve the primary goal of anesthesia—protecting the welfare of the animal when subject to invasive and potentially painful procedures. We also make empirically supported recommendations (see section Recommendations) for anesthesia and euthanasia during scientific procedures involving cephalopods.

### Definitions

Throughout the remainder of this paper we use the following definitions:

Anesthesia: “Anesthesia” is the collective term used commonly to refer to a multi-faceted effect on an animal: anterograde amnesia, anti-nociception, immobility, and loss of consciousness. Because we focus heavily in this study on blockade of afferent (sensory) signal, hereafter, we use the term “anesthesia” in its most literal and narrow sense; referring solely to the loss of sensation, which is represented experimentally as the absence of afferent neural signal produced in response to external stimuli.

Loss of efferent signal: Loss of conscious awareness during anesthesia is difficult to measure, even for human and mammalian patients. There is evidence that loss of consciousness is associated with decreased communication of neural signal originating in different regions of the brain (Purdon et al., 2013). Here, we use the operational definition that loss of consciousness may be inferred by the absence of organized, efferent neural signal. We note that “loss of consciousness” in human studies is often ascertained by behavioral measures such as absent responses to shouting at or shaking the patient (Villars et al., 2004); these are impractical for cephalopods, and complicated by the hypothesis that immobility in cephalopods occurs separately from loss of consciousness. Hence, we use loss of efferent signal as a proxy for loss of organized within-brain, and brain-body, communication.

Immobility: The complete absence of reflex or voluntary movements of the skin, arms, or body of the animal, not including respiration.

## Methods

### Animals

**Figure 1:**
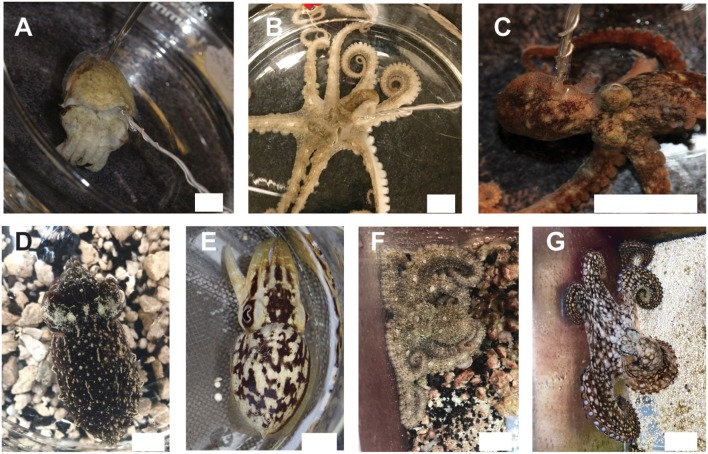
We studied effects of anesthesia on three tropical, commercially available cephalopod species. **(A)** *Sepia bandensis*. **(B)** *Abdopus aculeatus*. **(C)** *Octopus bocki*. Recordings were conducted *in vivo* using a minimally-invasive hook electrode, attached to one pallial nerve. Twenty-four hours after experiments, animals showed normal camouflage and signaling behavior **(D,E)** the same specimen of *S. bandensis* shown in **(A)**; **(F,G)** the same specimen of *A. aculaeatus* shown in **(B)**, demonstrating that neither the anesthesia nor the experimental procedure itself caused any long-term damage to the animal's nervous systems or behaviors. Scale bar: 15 mm.

Stumpy-spined cuttlefish (*Sepia bandensis*, Figure 1A) were reared from eggs purchased from a commercial vendor. Animals were between 3 and 5 months old (mantle length 25–40 mm) at the time of experiments. Cuttlefish were fed daily on live grass shrimp (*Palaemontes* spp.) and were housed in a recirculating, artificial seawater (Instant Ocean) system at 24.5°C. After experiments concluded, animals were returned to the housing system and remained in the lab until they died of natural causes. One cuttlefish was euthanized as part of the study. Cuttlefish were used in anesthesia trials once only.

Octopuses (*Abdopus aculeatus* and *Octopus bocki*, Figures 1B,C) were obtained as adults from commercial vendors, and were housed in individual enclosures visually isolated from other animals. Octopuses were fed 2–4 live shrimp on alternate days. Octopuses were either euthanized as part of the study, or remained in the lab at the conclusion of experiments and were later euthanized (according to the protocol described in this study). All animals were monitored daily for at least 3 days after experiments.

### Ethical note

In the USA, cephalopods are not considered animals for the purposes of animal welfare regulation, thus no protocol or approval number was required for this study. We followed the general guidelines for care and husbandry of cephalopods in the EU.

### Equipment

A hook electrode was made from insulated silver wire (0.15 inch diameter, Item AGTI510, World Precision Instruments, Sarasota, FL, USA), and the Teflon coating was stripped from the inner side of the hook. A ground of the same wire was wrapped around the shaft of the hook, and insulation stripped from the last 2 mm. The electrode was connected to an extracellular amplifier (A-M Systems model 2100), and electrical signal was sampled at 20 kHz and digitized by a PowerLab 4/35, while synchronized video footage of the experimental chamber was recorded using the “Video Capture” module of LabChart Pro software (AD Instruments). The recording chamber was a 125 mm diameter evaporation dish filled with 450 mL of artificial seawater taken from the home tank system. Equipment was identical for each of the species in the study.

### General procedure

Placing the hook electrode required animals to be sedated for handling, which was always done using ethanol in seawater. For cuttlefish, animals were placed immediately into 3% v/v EtoH in seawater, and remained undisturbed until they were behaviorally unresponsive to gentle disturbance. We did not observe any adverse reactions to the ethanol bath, which has been reported in some other studies (García-Franco, 1992). Once the subject was behaviorally unresponsive (typically within 3–5 min), the animal was held in a vertical positon and the mantle flap on the rostral edge of the mantle was moved aside, revealing the pallial nerve visible against the inner dorsal aspect of the mantle. Fine forceps were used to gently separate the nerve from the mantle, and the connective tissue was cut below the nerve with fine scissors. The hook electrode was passed behind the nerve, toward the caudal aspect of the mantle, then rotated and lifted to hook the nerve (Figure S1A). The hook was clamped loosely onto the nerve to provide good stability and contact between the exposed metal of the hook and the nerve. Desheathing the nerve was not necessary. The ground wire was positioned to sit freely from the nerve and muscle. No sutures or bonding material were needed to secure the electrode in place. At the conclusion of experiments, animals were re-sedated in ethanol and the hook was removed from the nerve by hand.

For octopuses, sedation in ethanol was conducted progressively, starting at 0.5% and increasing to 1, 2, then 3% in 5 min intervals. In octopuses, the pallial nerve is more anterior than in cuttlefish and is not connected to the mantle wall, thus the hook electrode was placed and clamped in position without the need to dissect away any connective tissues (Figure S1B).

Experienced experimenters were typically able to place the electrode in a cuttlefish in 2–3 min. In octopuses electrode placement took <1 min.

### Experimental procedure

As soon as the electrode was secured, animals were placed in the recording chamber, which contained artificial seawater taken from the main housing system (24.5°C) and recording of neural signal and behavior began. Gills and mantles were not irrigated with positive flow, so substances were exchanged from the gills by normal respiratory movements of the animals' mantles.

Every minute during anesthesia induction or reversal, we used grooved forceps to pinch the skin on the mantle in two locations (“medial,” located approximately over the stellate ganglion, and “distal,” on the ipsilateral rear tip of the animal's mantle), until the animal showed both behavioral and neural responses to pinch in both locations. Animals were judged to be completely recovered when they were upright, made coordinated swimming or jetting movements, showed normal chromatophore patterns, and showed normal responses to visual or other stimulation. Once animals had recovered from the initial sedation in ethanol, an experimental “general anesthetic” solution was added to the seawater bath, and the animal's induction process was monitored with distal and medial pinches each minute. In addition, we monitored changes in righting response, changes in spontaneous behaviors, changes in chromatophore patterning and loss of response to visual or other stimuli. We recorded the time between anesthetic induction and loss of spontaneous and evoked behavior responses, and latency to complete cessation of afferent and efferent neural signal evoked by pinch. At this point the solution was changed completely to normal seawater, and we monitored the recovery process using the same measures as described above. Most animals received two cycles of induction/recovery in the experimental anesthetic solution.

Once anesthesia was achieved on the second induction, one of two putative “local anesthetic” substances was injected subcutaneously on the distal mantle. A single bolus of 0.1 mL was injected with a 30G needle. We monitored behavioral and neural responses to pinch over the injected area until the animal was completely awake from the general anesthetic, then monitored animals' responses in their home tanks after the recording concluded.

Finally, we tested a standard euthanasia protocol. Once a single induction/recovery cycle of general anesthesia had concluded, animals were immersed in 330 mM MgCl_2_. Responses were monitored each minute, until 5 min after visible signs of respiration had ceased. While the recording continued, we incised the skin between the eyes, exposed the cranium, then used a scalpel blade to make multiple cuts through the central brain mass.

### Data analysis and statistical procedures

Electrophysiological signals were sampled at 20 kHz, amplified by a model 2100 extracellular amplifier (A-M Systems) and digitized using a PowerLab 4/35 (AD Instruments). Simultaneous recording of the neurophysiological signal and video recording of the behaving animal was obtained using the Video Capture model of LabChart Pro software (AD Instruments). Video was recorded at 60 Hz.

Files were scored by multiple experimenters, to check for agreement on scoring and to ensure impartial evaluation of data. From video data we recorded the latency from anesthetic substance introduction to the first behavioral sign of an effect, noted the time of other behavioral effect such breathing changes, loss of chromatophore control and loss of righting response, and latency until behavior had completely ceased. From neurophysiological traces, we recorded the latency to the complete absence of neural signal (i.e., only background electrical noise on the recording) evoked by pinch on the ipsilateral and contralateral side relative to the electrode.

Identification of afferent vs. efferent signal: On the ipsilateral side to the electrode, signal recorded was likely a combination of afferent and efferent spikes, and as such we did not always make determinations of signal direction. Pinches on the contralateral side produced signal on the recorded trace that were considered to be purely efferent, as signal leaves the brain through the paired pallial nerves to produce coordinated responses on both sides of the mantle, and afferent signal does not cross the midline of the body before entering the brain. In cases where there were no spikes evoked by contralateral pinch, but there were spikes evoked by ipsilateral pinch, we considered that this activity was most likely to be afferent signal only. Loss of efferent signal is indicated in the temporal sequences of neural activity for cuttlefish (**Figures 5A,B**) and octopuses (**Figures 6A,B**).

#### Statistical analyses

Latencies were normally distributed and were analyzed with parametric statistics. We used paired *t*-tests to test for significant differences between the latency from drug onset to loss of evoked behavioral response, and from drug onset to loss of afferent signal (anesthesia) upon induction, and between return of neural signal and return of behavioral responses upon reversal. Independent sample *t*-tests were used to compare behavioral or neural metrics between the two anesthetic substances, within species. Lags and advances (time difference between neural and behavioral signal loss or recovery) were tested with one-sample *t*-tests with expected values of zero. We used a critical α of 0.05, and all *p*-values reported are two-tailed.

## Results

### *In vivo* recording of peripheral nervous system activity

**Figure 2:**
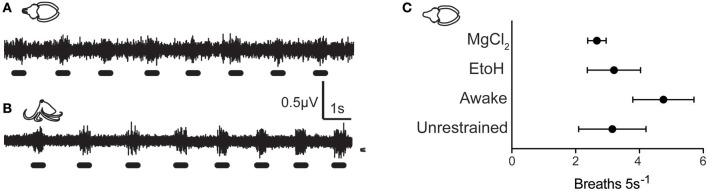
Background neural activity during quiescent, awake periods of cuttlefish **(A)** and Octopus (*O. bocki*, **B)**, showing rhythmic bursts associated with mantle contractions during respiration. Respiration rates during anesthesia can be computed from electrophysiological traces, and respiration signal was a good indicator that the electrode was well-positioned, the nerve was healthy, and that animal was physiologically stable. **(C)** Respiration rates of animals in various procedure stages did not differ significantly. Awake animals showed slightly elevated rates of respiration which was likely due to handling stress. Respiration rates from cuttlefish in the EtoH and MgCl_2_ treatment groups were measured during periods in which no afferent neural signal was detected during mantle pinch. Breathing rates were not significantly slower than during awake periods, and were within the range of normal respiration rates of unrestrained animals observed in home tanks.

All animals in the study tolerated the hook electrode well (Figures 1A–C), with only one cuttlefish showing signs of nerve damage after hook removal (ipsilateral mantle paling). Twenty-four hours after testing, all other animals showed normal cryptic and signaling behavior (Figures 1D–G) indicating that the procedure produced no adverse effects on the nervous system or skin. Signal recorded by the electrode included afferent and efferent spikes ranging in amplitude from 0.2 to ~3 μV, and was qualitatively similar among the three species. Variation in electrode-nerve contact caused substantial between-trial variation in spike amplitudes, although overall patterns were similar. Results from the two species of octopus were pooled. In addition to capturing evoked responses to touch, we also recorded spontaneous activity in the nerve associated with chromatophore activity, mantle contraction during swimming, and ventilation. Example signal from respiratory motor neurons in fully awake cuttlefish and octopus is shown in Figures 2A,B, respectively. Respiration rates were not significantly slower during periods of complete anesthesia (absence of evoked afferent signal) compared with awake periods, or with rates of unrestrained animals in home tanks.

### Effective doses

**Figure 3:**
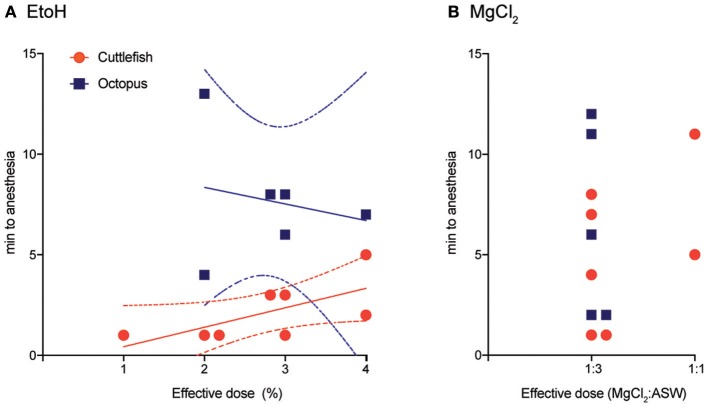
Effective doses and the time to anesthesia (the loss of neural signal at both the medial and distal stimulation sites). **(A)** Effective doses of ethanol ranges from 1 to 4% in ASW. There was a weakly positive correlation between does and time-to-induction for cuttlefish (Pearson *r*^2^ = 0.89, *p* = 0.02), line shows best fit from linear regression, dotted lines are 95% CI), but no relationship for octopus. **(B)** Nearly all subjects were effectively anesthetized in a solution of 1:3 MgCl_2_: ASW, but times to anesthesia varied considerably. There was insufficient variation in dose to test dose/time relationships.

Effective doses (those that clearly affected both behavior and neural signal in fewer than 5 min from introduction) varied from 1 to 4% for cuttlefish and octopuses exposed to ethanol, and from 1:3 to 1:1 for MgCl_2_:ASW (Figures 3A,B). Effective doses were determined on the first induction of each experimental trial by progressively increasing the dose (in 1% increments for ethanol and from 1:3 to 1:1 mixes for MgCl_2_) until an effect was apparent. On the second induction for each trial, the effective dose was administered directly, without progression, and the data averaged within animal. In general, effective dose was weakly positively correlated with induction times for cuttlefish under ethanol anesthesia (Pearson *r*^2^ = 0.89, *p* = 0.02), suggesting that some animals were more resistant to anesthesia than others, requiring both higher doses and longer times to anesthesia. There was no pattern for octopuses. Nearly all animals tested were effectively anesthetized by a 1:3 mix of MgCl_2_ in ASW.

### Induction

**Figure 4:**
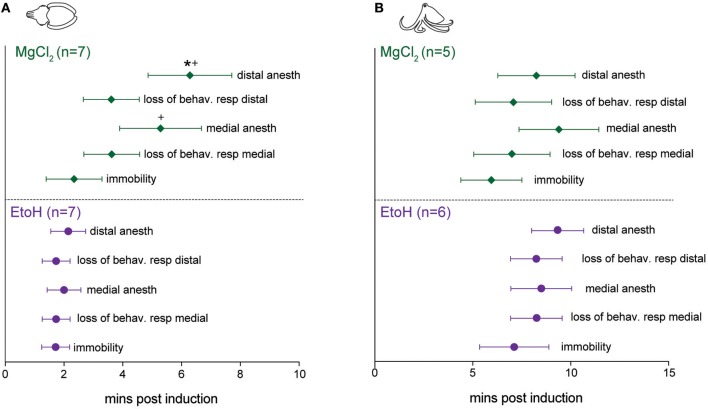
Latencies to behavioral and neural indicators of tri-partite anesthesia induction for cuttlefish and octopus (*O. bocki* and *A. aculeatus* pooled). **(A)** Ethanol anesthesia (effective doses pooled) showed tight temporal coupling of immobility, loss of evoked behavioral responses and loss of evoked neural responses. While magnesium chloride achieved the three requirements of anesthesia—immobility, loss of afferent signal and loss of efferent signal (not shown on figures, see Results for details)—there was a significant delay between the loss of behavioral response and the loss of neural response at the distal stimulation site (denoted by ^*^). Latencies to medial and distal anesthesia were significantly delayed compared with the same measures for ethanol anesthesia (denoted by +). **(B)** The same variables shown for octopuses. Octopus sample sizes were smaller, and octopuses were inherently more variable, thus although patterns are similar to those of cuttlefish there are no significant differences. Within-treatment comparisons: paired *t*-tests. Between treatment comparisons: unpaired *t*-tests. All *p*-values are two-tailed. ^*^p < 0.05.

**Figure 5:**
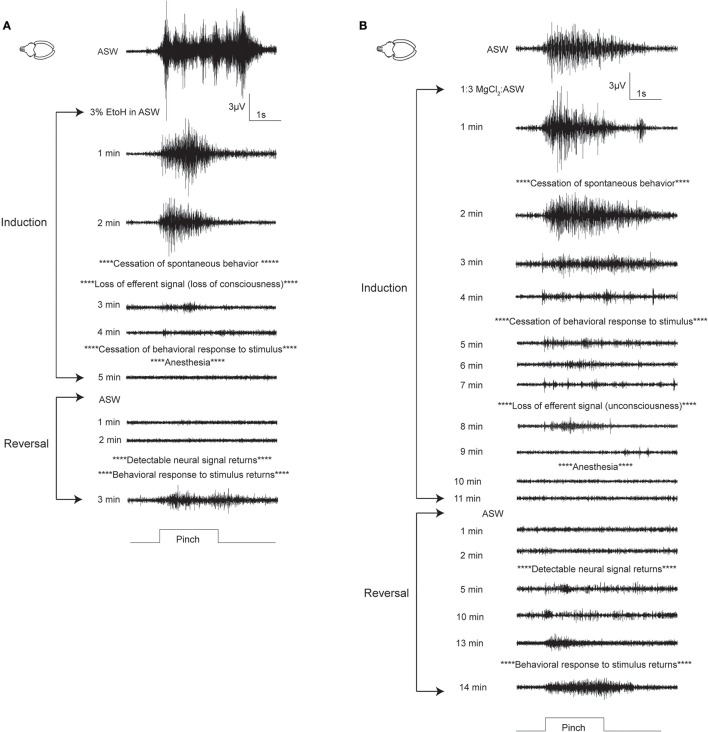
Examples of electrophysiological traces recorded during pinch on the distal stimulations site, for cuttlefish in ethanol **(A)** and MgCl_2_ **(B)**. **(A)** A fully-awake cuttlefish has a strong response to pinch prior to anesthesia induction. This trace shows motor efferent signal associated with escape jetting (this animal makes 7 jets in rapid succession, coinciding with peaks in the signal). After 2 min in 3% ethanol, the animal ceases spontaneous behavior, and there is no neural signal in response to pinch on the contralateral side to the electrode (trace not shown). Evoked behavior (which may be reflexive or intentional) ceases after 4 min, at the same time that neural afferent signal ceases. Reversal in ASW is rapid, with simultaneous return of evoked behavioral and neural signals. **(B)** Cuttlefish undergoing MgCl_2_ anesthesia showed a different pattern. While spontaneous and evoked behavior ceased at a similar time to the animal in ethanol in these examples, afferent and efferent neural signal persisted for much longer, and their loss was temporally more separated than for ethanol. Evoked afferent signal in response to pinch was present for more than 10 min after the cessation of evoked behavioral responses; this period is of great concern for welfare. Upon reversal, neural signal returned rapidly, but there was a length period in which behavioral responses remained absent; again, the existence of this extended period is likely to be problematic for researchers using behavioral measures to ascertain anesthesia. Fully awake behavior usually lagged behind return of evoked behavioral responses by more than 5 min.

Patterns of induction were similar in cuttlefish exposed to magnesium chloride (*n* = 7, Figure 4A top, Figure S2) and ethanol (*n* = 7, Figure 4A bottom, Figure S3), although there was more spread in latencies for magnesium chloride. Behavioral and neural indicators of induction had highly similar latencies from the introduction of ethanol, with no significant differences between any of the measures. Latencies from introduction of magnesium chloride to behavioral signs of induction (immobility, evoked responses to pinch) did not differ from those in ethanol, but loss of neural signal took significantly longer compared with ethanol (medial: unpaired *t*-test, *p* = 0.049, distal: *p* = 0.02). There was also a significant delay between the loss of behavioral response to distal pinch and the loss of neural signal in response to distal pinch (paired *t*-test, *p* = 0.015). Examples of traces from a single sequence of induction and recovery from ethanol anesthesia are shown in Figure 5A. Induction was characterized by a progressive decline in signal, with a clear decrement observed at the point where spontaneous behavior and efferent signal ceased. Anesthesia (complete loss of afferent signal) followed rapidly thereafter. In contrast, traces from a single induction and recovery cycle for a cuttlefish exposed to magnesium chloride (Figure 5B) show a prolonged period where afferent and efferent signal persist with minimal progressive deficit (from 4 to 8 min), after cessation of behavioral responses and prior to complete anesthesia.

**Figure 6:**
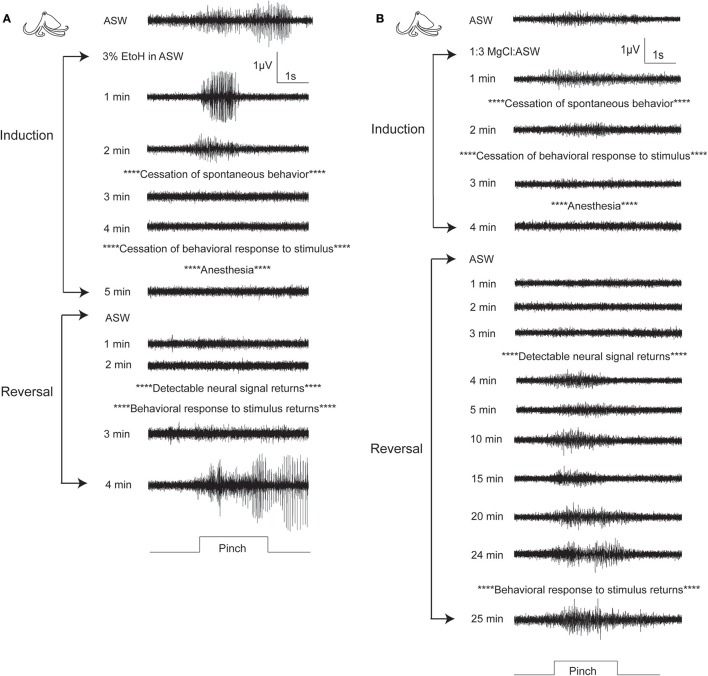
Examples of electrophysiological traces recorded during pinch on the distal stimulation site, for one octopus undergoing ethanol **(A)** or MgCl_2_ **(B)** anesthesia. **(A)** A fully awake octopus shows strong neural response to pinch. Within 2 min of induction in ethanol, spontaneous behaviors have ceased and neural signal is almost completely abolished. By 5 min there is no neural and no behavioral responses to pinch. Similar to cuttlefish under ethanol anesthesia, reversal is rapid with simultaneous return of neural and behavioral responses to pinch. **(B)** In this example of an octopus in MgCl_2_, anesthesia induction is uncharacteristically rapid (compare with means shown in Figure 4), with minimal delay between loss of behavioral responses and loss of neural responses. We selected this example to show the very prolonged recovery process that was typical of octopuses anesthetized in MgCl_2_. By 4 min after reversal begins, neural signal is apparent, and it remains strong and mostly unchanging for the next 20 min. There is a slight increase in signal amplitude and duration at 24 min, and the first behavioral response to pinch is recorded at 25 min after reversal begins. During this period there is no behavioral indication that the animal's nervous system is functional. Behaviors indicating that animal is fully awake typically returned 5–10 min after evoked behaviors.

For octopuses, (Figure 4B, Figures S4, S5), patterns were similar to those seen in cuttlefish, although octopuses generally took longer to show effects. There were no significant differences in any behavioral or neural measure either within or between substances. Examples of traces from a single sequence of induction and recovery from ethanol anesthesia are shown in Figure 6A. Similar to cuttlefish, octopuses in ethanol showed a rapid loss of neural signal that was associated with cessation of spontaneous behavior, and reached full anesthesia at the same time as evoked behavioral responses ceased. Traces from octopuses in MgCl_2_ (Figure 6B) showed a similar induction process, except that loss of evoked behavioral response preceded anesthesia.

### Reversal

**Figure 7:**
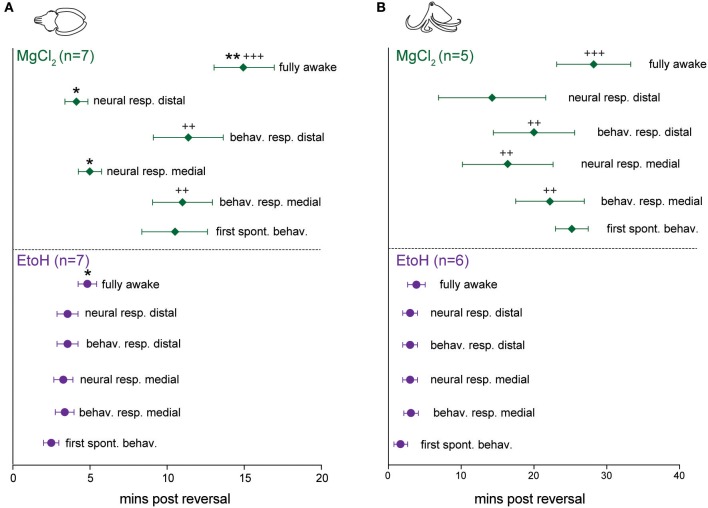
Latencies to behavioral and neural indicators of anesthesia reversal for cuttlefish and octopus (*O. bocki* and *A. aculeatus* pooled). **(A)** Cuttlefish undergoing reversal of ethanol anesthesia (**A**, bottom) showed tight temporal coupling of return of spontaneous behaviors, return of evoked behavioral responses and return of evoked neural responses. Return of neural signal in cuttlefish under magnesium chloride anesthesia (**A**, top) occurred on a similar timescale to that for ethanol, but return of behavioral responses was significantly delayed, both with respect to neural signal in the animals (denoted by ^*^), and with respect to behavioral recovery for ethanol (denoted by +). **(B)** The same variables shown for octopuses. **(B)**, bottom: Octopuses undergoing reversal from ethanol anesthesia showed tight temporal coupling of all reversal indicators. Octopuses undergoing reversal of MgCl_2_ anesthesia showed significant delays of neural responses at the medial stimulation site, behavioral responses at both sites, and latency to full recovery compared with ethanol-treated octopuses, but within-group, data were highly variable and not significantly different. Within-treatment comparisons: paired *t*-tests, marked with ^*^. Between treatment comparisons: unpaired *t*-tests, marked with +. All *p*-values are two-tailed. ^*^p < 0.05, ^**^p < 0.01, ^***^p < 0.001.

Reversal of anesthesia for cuttlefish in ethanol (*n* = 7, Figure 7A bottom) was characterized by rapid, highly consistent and statistically simultaneous return of spontaneous behavior, neural responses to pinch and evoked behavior in response to pinch. There was a brief but significant delay between the first incidence of evoked neural signal and the animals showing a “fully awake” state (paired *t*-test, *p* = 0.013). Cuttlefish in magnesium chloride (*n* = 7, Figure 7A top) showed return of neural signal at similar times to those animals exposed to ethanol, but behavioral responses were significantly delayed both in comparison to neural signal in the same animals (paired *t*-tests: medial *p* = 0.032, distal *p* = 0.03, and in comparison to behavioral signs of recovery after exposure to ethanol (unpaired *t*-test: medial, *p* = 0.005, distal, *p* = 0.0015). There was also a significant delay from the first incidence of evoked neural signal and the animals showing a “fully awake” state (paired *t*-test, *p* = 0.0035), and between the latency to show fully awake behavior compared with animals in the ethanol group (unpaired *t*-test, *p* = 0.0009).

For octopuses in the ethanol group (*n* = 6, Figure 7B, bottom), trends and latencies were similar to those seen in cuttlefish, except that there was no significant delay between the first incidence of evoked neural signal and fully awake behaviors. An example of electrophysiological traces from an octopus undergoing reversal of ethanol anesthesia are shown in Figure 6A. In the magnesium chloride group (*n* = 5, Figure 7B top), latencies were highly variable for all measures, with no significant differences among any of the measures within the group. Return of behavioral responses to medial and distal pinches, return of evoked neural activity in response to medial pinch, and latency to being fully awake were all significantly delayed compared with measures for the ethanol-treated octopuses (unpaired *t*-tests, medial behavior: *p* = 0.004, distal behavior: *p* = 0.002, medial neural: *p* = 0.02, fully awake: *p* = 0.003). Although not significant, octopuses treated with magnesium chloride tended to recover spontaneous behaviors later than evoked behaviors, a pattern not seen under ethanol treatment for either cuttlefish or octopuses, or in the magnesium chloride group for cuttlefish.

The latencies between the loss of evoked behavioral responses and evoked neural responses, we hereafter term “neural lag.” This lag is the interval where an animal may appear to have entered a true anesthetized state (that is, where afferent signal is absent), but is in fact simply immobilized. The inverse relationship upon reversal (i.e., where neural signal returns prior to behavioral responses), we term “neural advance.” This advance is the interval where the animal appears to still be anesthetized, but is not. These two periods represent critical points during invasive procedures where the animal's welfare may be compromised.

**Figure 8:**
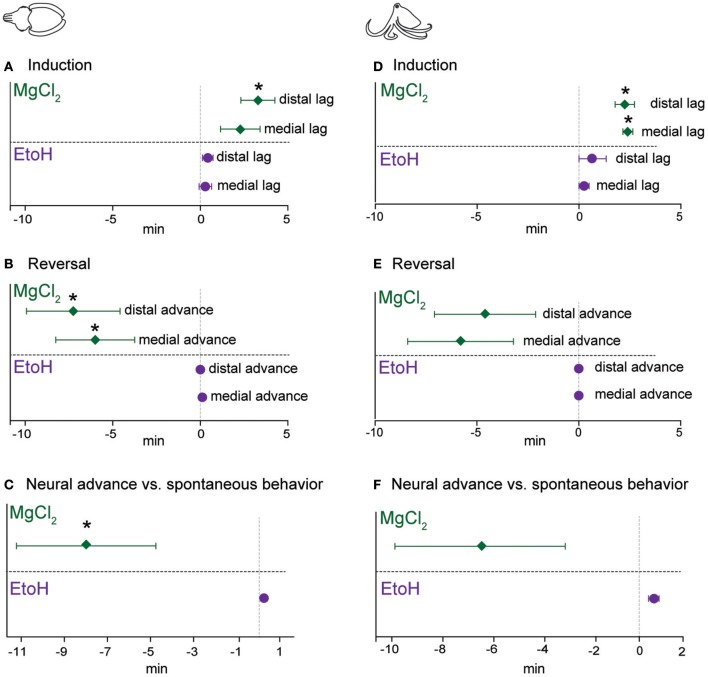
Lag of neural signal loss (compared with behavior) upon induction, and advance of its return (compared with behavior) upon reversal. For each experimental trial, we used the following formula to compute “neural lag” (for induction) and “neural advance” (for recovery). Lag or Advance = (latency to loss/return of neural signal—latency to loss/return of behavioral signal). Thus, a positive value upon induction shows that neural signal persisted longer than behavioral responses did, and a negative value upon reversal shows that neural signal returned sooner than behavioral responses returned. Each outcome is tested with a one-sample *t*-test against an expected value of zero (i.e., neural and behavioral responses are lost or return simultaneously) **(A)** Cuttlefish induced with ethanol had very little very lag, while for those in magnesium chloride group, there was a significant lag at the distal site. Upon reversal **(B)**, cuttlefish in the ethanol group had advances of zero, while both stimulation sites showed significant neural advances for cuttlefish in the magnesium chloride group. We also compared the advance of neural recovery compared with the return of spontaneous behavior **(C)**, here also there was effectively zero advance for ethanol but a significant advance for magnesium chloride. Patterns were largely similar for octopuses. **(D)** Both the medial and distal stimulation sites showed significant neural lags for octopuses in the magnesium chloride group, and although there was some slight lags for the ethanol group, neither was significantly different from zero. **(E)** Upon reversal, there were noticeable but non-significant advances (medial; *p* = 0.08, distal, *p* = 0.13) at both stimulation sites for the magnesium chloride group, but effectively zero advances for the ethanol group. **(F)** Advance of neural recovery compared with the return of spontaneous behavior for octopuses was significant (*p* = 0.002, note that this is in the positive direction, i.e., spontaneous behavior occurred sooner than neural recovery, see results for explanation). There was a long advance for octopuses in the magnesium chloride group, but this was not significantly different from zero (*p* = 0.12), most likely due to the highly variable advances across trials. ^*^p < 0.05, ^**^p < 0.01, ^***^p < 0.001.

For cuttlefish induced with ethanol (Figure 8A), neural lags upon induction were negligible, but were slightly positive (i.e., a positive value indicates behavioral signal is lost earlier than neural signal) for magnesium chloride. There was a significant lag in anesthesia onset at the distal pinch location (one-sample *t*-test vs. 0, *p* = 0.014). During reversal of ethanol anesthesia, neural signal return was not advanced compared with behavioral signal (Figure 8B). In contrast, advances were significantly different from zero for magnesium chloride anesthesia in both test locations (a negative value on the graph indicates neural signal returns earlier than the behavioral signal; medial, *p* = 0.032, distal, *p* = 0.03). Lastly, we plotted the advance of neural signal compared with the re-appearance of spontaneous behaviors (Figure 8C), as a measure of the period between when afferents in either location regain their function and when the nervous system, as a whole, regains its function. For ethanol this was almost simultaneous; deviations from zero are an artifact of the test procedure because we noted spontaneous behaviors at any time, but only tested neural signal each minute. In contrast, there was a significant advance of evoked afferent signal in comparison to the re-appearance of spontaneous behavior for cuttlefish in the magnesium chloride group (*p* = 0.048).

Patterns were similar for octopuses. During induction (Figure 8D), there were non-significant lags between disappearance of evoked behaviors and evoke neural activity for ethanol, but both medial (*p* = 0.006) and distal (*p* = 0.012) stimulation sites showed significant lags in the magnesium chloride group. Upon reversal (Figure 8E), there was no advance in the reappearance of neural signal compared with reappearance of behavioral responses to pinch, at either location, for ethanol anesthesia. In the magnesium chloride group, there were substantial but non-significant advances in the return of neural signal compared with the return of evoked behavioral responses. Similarly, there was a substantial but non-significant advance of evoked afferent signal in comparison to the re-appearance of spontaneous behavior for cuttlefish in the magnesium chloride group (Figure 8F).

### Behavioral signifiers of induction and reversal

**Figure 9:**
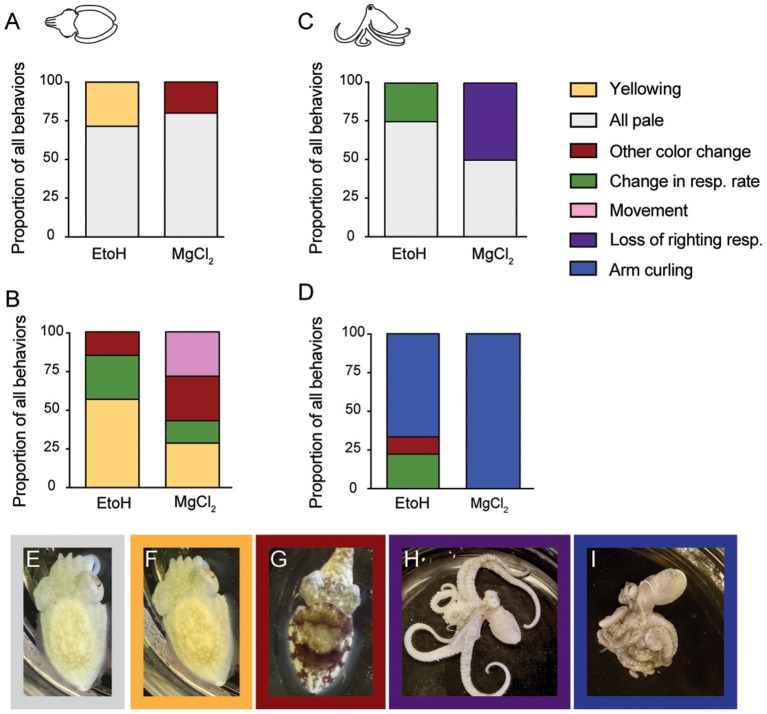
Behavioral signifiers of anesthesia induction and reversal. **(A)** Cuttlefish undergoing ethanol induction most frequently showed an all-pale **(E)** body color as their last spontaneous behavior before loss of neural signal. All-yellow **(F)** was also common for ethanol, and chromtophore pulsing, flickering, or waving occurred in some animals in the MgCl_2_ group. Upon reversal of anesthesia **(B)**, cuttlefish in the ethanol groups showed all-yellow most frequently as the behavior occurring closest to re-emergence of neural responses to pinch. Other behaviors included increased respiration rate (see Figure 2 for example), and other color changes **(G)**. In the MgCl_2_ group, behavioral indicators were more varied, with all-yellow, other color change, movement, and increased respiration noted as the behavior occurring closest to return of neural signal. For octopus inductions **(C)**, all-pale was the most common behavioral sing of anesthesia in both ethanol and magnesium chloride groups. Respiration rate change in the ethanol group and loss of righting response **(H)** in the magnesium chloride group were also frequent indicators. Reversal for octopuses **(D)** was reliably signaled in both groups by a sudden coiling or curling of the arms **(I)**. In the ethanol group, respiration rate changes and color change were also noted.

We categorized behaviors that were most closely linked temporally (i.e., the last noted behavioral change before neural signal ceased) with the onset of anesthesia and of anesthesia reversal (the behavioral that first occurred either right before or after return of neural signal), for each substance and for each taxon (Figure 9). For cuttlefish undergoing ethanol anesthesia (Figure 9A), sudden, whole-body paling (Figure 9E) was the most frequently occurring behavior in the minute before loss of neural signal was noted. Less frequent was an all-yellow color (Figure 9F) In general, onset of ethanol anesthesia was reliably indicated by relaxation of chromatophores. For cuttlefish undergoing magnesium chloride anesthesia (Figure 9A), all-pale was also the most frequent indicator of anesthesia. Occasionally, we noted chromatophore flickering, waving or pulsing instead of paling; we group these various changes under “other color change.”

Behavioral indicators of reversal for cuttlefish (Figure 9B) were more variable than for induction. The most frequent behavior in both the ethanol and magnesium chloride groups was yellowing (Figure 9F). We also observed some animals show a sudden and pronounced change in respiratory rate, or show a strong mottle color pattern (Figure 9G). In the magnesium chloride group, spontaneous arm or fin movement was noted in some animals. However, we note that in most cases in the magnesium chloride group, the first behavioral indicator of reversal appeared *after* return of neural signal.

For octopuses, induction with either agent was most frequently signaled by relaxation of chromatophores (paling) (Figure 9C). In the ethanol group, the other indicator was a sudden decrease in respiration rate. For magnesium chloride, loss of righting response (Figure 9H) was the best indicator of induction in about half of all trials. Reversal of anesthesia in octopus was indicated in almost all trials by a very distinct and abrupt tight coiling of all the arms (Figures 9D,I). This was the universal indicator of reversal for magnesium chloride, but as for cuttlefish, we note that this first behavioral indicator of reversal appeared *after* return of neural signal. For the ethanol group, a sudden increase in respiratory rate or other color change (sudden reappearance of strong mottled pattern) were the other indicators.

### Unsuccessful general anesthetic agents

We tested a small number of animals with other candidate anesthetic agents: chilled (4°C) ASW (*n* = 2 cuttlefish), 1% diethyl ether in ASW (*n* = 2 octopus, *n* = 1 cuttlefish), and 500 mg/L MS-222 (tricaine) in ASW (*n* = 1 cuttlefish). In chilled seawater, initial exposure produced sustained, high frequency firing (Figure S6) and behavioral signs of aversion, including strong withdrawal of the head into the mantle and escape jetting. Although neural signal was lost after around 5 min and animals recovered well in warmed seawater, we concluded that the procedure was likely aversive.

Animals tested in 1% ether showed rapid induction, with all losing neural signal within 2 min of exposure. No animals showed signs of aversion or stress upon induction, however, reversal times were extremely lengthy, characterized by relatively rapid return of afferent signal and evoked behavior, but no further recovery toward normal, spontaneous behavior. One animal showed impaired vestibular function for multiple hours after return to the home tank, but thereafter appeared normal.

We attempted MS-222 general anesthesia on one cuttlefish only. There is no suggested dose for cephalopods, so we first tried 100 mg/L, the standard dose for general anesthesia of fish. This was ineffective, and the animal produced strong escape jetting in response to its introduction. After 2 min, we increased the dose to 500 mg/L, half the effective dose recommended for amphibians. The resulted in respiratory arrest 7 min after introduction, and no apparent effect on afferent neural signal. Despite a lengthy period of manual respiration in aerated, flowing seawater, the animal did not recover.

### Local anesthesia

**Figure 10:**
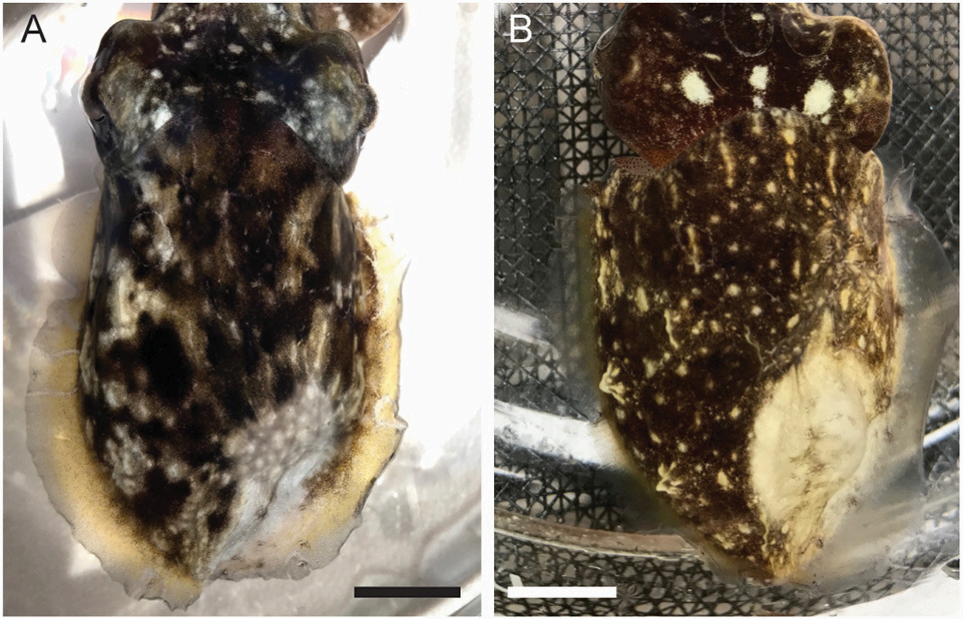
Representative images of the distal mantle of a cuttlefish injected with **(A)**. 0.1 mL of 0.5% lidocaine in ASW, and **(B)**. 0.1 mL of isotonic (330 mM) magnesium chloride. Images taken 20 min after injection. Scale bars 10 mm.

We tested two candidate local anesthetic drugs on cuttlefish; 0.1 mL lidocaine (0.5%, *n* = 5), and 0.1 mL isotonic magnesium chloride (*n* = 4). Both drugs provided highly effective blockade of sensation, with average time to complete absence of evoked neural signal of 3 min 20 ± 46 s for lidocaine, and 2 min 15 ± 38 s for isotonic magnesium chloride (unpaired *t*-test, *p* = 0.34). Absence of neural signal persisted until recordings were completed (11 ± 4 min after injections, *n* = 9), but we did not test durations beyond. Both drugs produced changes in chromatophore activity over the area of infiltration (Figure 10).

### Euthanasia

**Figure 11:**
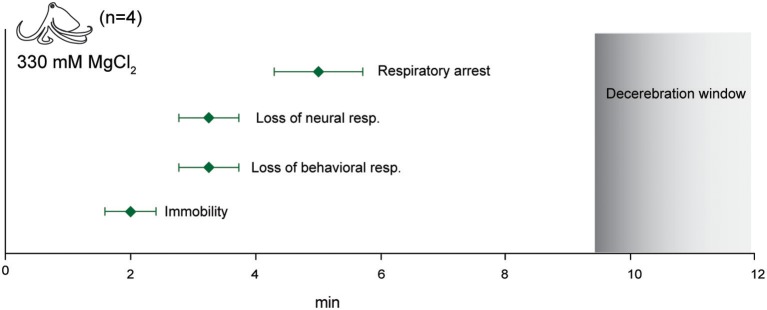
Euthanasia. Immersion in isotonic (330 mM) magnesium chloride resulted in rapid loss of afferent and efferent signal and respiratory arrest. No neural signal was recorded upon skin and cranial incision 5 min after respiratory arrest. This interval is shown as the “decerebration window” where the animal is deeply anesthetized and insensible during destruction of the CNS.

We tested four octopuses and one cuttlefish using a standard euthanasia protocol—immersion in 330 mM MgCl_2_ followed by decerebration (Figure 11). Octopuses took around 5 min until complete respiratory arrest, but this was often preceded by 1–2 min of extremely slow respiration of around 3–4 breaths per minute. Five minutes after breathing had ceased (and an average of 7 min after neural signal had ceased), we began decerebration while monitoring signal on the pallial nerve. We observed no behavioral, reflexive or neural responses to skin incision or to surgical destruction of the brain.

## Discussion

Here, for the first time, we provide conclusive evidence that both ethanol and magnesium chloride are effective anesthetic agents for three species of tropical cephalopods. We expect that these results should be applicable to many other cephalopod species, thus providing new assurance to researchers, institutional animal care bodies and legislative agencies charged with protecting the welfare of cephalopods in research. Although the species that we tested were tropical, we anticipate that effective anesthesia in temperate species should be readily achievable using these methods.

The two substances differed markedly in the temporal characteristics of their effects. Induction and reversal in ethanol was rapid and was indicated reliably by behavioral changes that were tightly temporally coupled with loss and return of afferent neural signal. In contrast, anesthesia in magnesium chloride was considerably delayed compared with immobility. Even though neural signal was blocked reliably in all trials, there were no behavioral indicators that were tightly coupled with anesthesia. Thus, unless researchers are prepared to monitor neural signal directly, ascertaining anesthesia depth with magnesium chloride anesthesia is likely to be almost impossible.

Magnesium chloride and ethanol are generally not accepted anesthetic substances for any taxon other than cephalopods; as such, there is limited knowledge of their mechanism of action in promoting anesthesia-like states. Magnesium chloride is generally considered to be a muscle relaxant or sedative in vertebrates (Sung et al., 2017), but there is some evidence that is also anti-nociceptive (Kroin et al., 2000; Albrecht et al., 2013; De Oliveira et al., 2013). Ethanol also has analgesic properties and can certainly affect consciousness and produce amnesia in vertebrates (Tamerin et al., 1971; Messing, 2014; Thompson et al., 2017), but its molecular targets or mechanisms in cephalopods are not clear. Despite their differing effects and uses in more typical vertebrate models of anesthesia, for both substances we show conclusively that while they are certainly effective at producing immobility and sedation, they are also effective at blocking both afferent and efferent neural signal. This effect does not appear to be due to hypoxia resulting from respiratory depression (although we note that we did not monitor hemolymph oxygen content, and this cannot completely exclude the possibility that hypoxia contributes to the anesthetic effects we observed) or from damage to the neurons themselves; thus, in cephalopods at least, these agents act as “functional” anesthetics that are readily reversible and from which animals recover with no apparent ill effects.

We also show that several other agents are unsuitable as general anesthetics for cephalopods, although they have utility in other species. We had hypothesized that diethyl ether, a volatile anesthetic with many shared molecular targets to ethanol (Solt and Forman, 2007), would show induction and reversal characteristics similar to ethanol. While the induction times for ether were similar, reversal was protracted; evidently the unbinding of ether molecules from one of its molecular targets is significantly delayed in cephalopods. In addition to producing delayed reversal times, ether can be hazardous to laboratory personnel and requires special handling, making it impractical as well as unsuitable. We also hypothesized that tricaine methanesulphonate (MS-222), a blocker of voltage-gated sodium channels (Attili and Hughes, 2014; Ramlochansingh et al., 2014), would act similarly in cephalopods as in vertebrates due to the ubiquity of voltage-gated sodium channels in nervous tissue throughout the animal kingdom. Instead, we found no anesthetic effect at a low dose and a lethal effect at an intermediate dose, although this was tested only on one animal.

We tested cold water as an anesthetic agent because it had been reported to be an effective adjuvant to other substances in some studies (Andrews and Tansey, 1981), and because it has been used as a euthanizing method in others (Staudinger et al., 2011). The strong behavioral and neural evidence we observed for its aversiveness (Figure S5) indicate that it is not suitable for either anesthesia or euthanasia in tropical species, although we do not rule out that it may be suitable for temperate cephalopods. Further work is needed to identify the precise molecular targets of each of these substances in cephalopods, and to determine their binding kinematics.

While we were successful at demonstrating three aspects of general anesthesia, we did not tests the fourth component, anterograde amnesia (Villars et al., 2004). Cephalopods are highly capable of many different learning and memory tasks, and multiple species show excellent performance in habituation (Agin et al., 2006a; Kuba et al., 2006), sensitization (Crook et al., 2011; Alupay et al., 2014), classical conditioning (Cole and Adamo, 2005; Crook and Basil, 2008) and operant conditioning (Darmaillacq et al., 2004, 2008; Agin et al., 2006a,b; Alves et al., 2007; Zepeda et al., 2017) procedures. We suggest that these validated learning procedures that produce short-term memory after a single training trial could be modified for such a test.

We also tested local anesthesia, which we used in conjunction with general anesthesia in this study for ease of application and to reduce handling stress. While it is possible that some interaction between general and local anesthetics occurred, it is clear that local anesthesia is readily achievable by subcutaneous or intramuscular injection in cephalopods. Local anesthesia use has been reported infrequently in cephalopods, although unpublished observations of the efficacy of several candidate substances (xylocaine, mepivicaine) have been reported (Fiorito et al., 2015), and complete nerve block by isotonic magnesium chloride has been shown previously (Crook et al., 2013, 2014). Lidocaine has been shown to have adverse effects on cultured neurons of gastropods (Onizuka et al., 2005, 2012a,b), but we observed no obvious long-term effects in the animals we tested, however, the injection location in our study would have affected only the peripheral terminals of sensory neurons, and different or adverse results may be observed by applying lidocaine to ganglia.

Cephalopods with localized tissue injury show both local and generalized sensory neuron hyperexcitability after injury (Crook et al., 2013; Alupay et al., 2014; Perez et al., 2017) that is activity dependent; silencing of sensory afferents during injury with any effective local anesthetic agent should therefore produce both local anesthesia and some measure of generalized or systemic analgesia. Although we did not measure the duration of local anesthetic effects in the study, we are confident that they extend beyond the period of general anesthetic reversal. Thus, an animal that has undergone a surgical procedure under general anesthesia without local anesthesia may experience immediate, strong activation of now-sensitized (or spontaneously active) nociceptive afferents (Crook et al., 2013; Perez et al., 2017) immediately upon reversal of general anesthesia. In contrast, cephalopods that received an injectable local anesthetic at the surgical site may have a considerably prolonged period of relief from sensitized nociceptive input. In the absence of validated analgesic agents for cephalopods, applying local anesthetic agents at the time of issue injury is likely to be a meaningful way of promoting welfare.

We tested one method of euthanasia only, although multiple methods have been reported (Andrews et al., 2013). The procedure we used produced rapid anesthesia and loss of consciousness (no efferent signal), and respiratory arrest that followed rapidly thereafter. We did not monitor heart function directly, therefore it is possible that the heart continued to beat for some period after respiration ceased. We also monitored neural signal only in the peripheral nervous system. Although we observed no neural signal in the pallial nerve during the decerebration procedure, we cannot exclude the possibility that some residual neural activity in the central brain was ongoing at the time of decerebration, or that incision into the brain produced a burst of activity that was not represented in efferent output. We hypothesized that if the animal were consciously aware of the incision either into the skin of the head or into the brain itself, we would have observed activity in the pallial nerve, which carries motor neuron signals to muscles of the mantle that generate escape behavior. However, further study of central brain activity during euthanasia is needed.

### Recommendations

#### General anesthesia

Both ethanol and magnesium chloride are “functional” anesthetics in cephalopods. Ethanol anesthesia is likely to be easiest to manage; the tight and reliable relationship between behavioral responses and peripheral, afferent neural signal should allow researchers clear indications of depth of anesthesia without the need to monitor neural signal directly. Induction with ethanol was rapid and appeared to cause no adverse behavioral effects. Reliable behavioral indications of sufficient depth are easily observed. At the point where these external signifiers of awareness cease, surgery or other invasive procedures may occur with assurance that the animal's welfare is protected. Upon reversal of ethanol anesthesia, changes in efferent signal typically lagged behind changes in afferent signal and coincided with behavioral responses, thus monitoring behavioral responses such as increased respiration depth, change in color from uniform gray to uniform yellow, and observing the whole body carefully for movement responses to stimulation, should provide good indication of reversal.

We make special note that in octopuses in particular, reversal of ethanol anesthesia may be spontaneous, with breakthrough behavior occurring without changes in the anesthetic concentration; but we found that this did not occur in cuttlefish. Careful monitoring of octopus is necessary, and we recommend that local anesthetic be employed around surgical sites in combination with general anesthesia, prior to incisions being made.

While this relationship is less reliable under magnesium chloride anesthesia we suggest that for some procedures, the long recovery times and better muscle relaxation we observed in this study might allow for extended surgical procedures without the need for additional anesthesia dosing. In no circumstances do we recommend cold seawater, ether or tricaine be used as anesthetic or sedating agents for tropical species.

#### Local anesthesia

Both local anesthetics we tested showed very good reliability and efficacy, with no apparent adverse effects on tissue integrity or normal function after recovery. Magnesium chloride was effective at blocking sensation, and the strong muscle relaxant effect provides an easily observed read-out of the infiltration's extent; this paling was less clearly defined, although still present, with lidocaine. However, we caution that the muscle relaxant properties of magnesium chloride make it unsuitable for use at surgical sites. Lidocaine also produced good blockade of sensation, however its infiltration margins were less apparent than for magnesium chloride. Wide injection margins around surgical sites should be used wherever possible. Its less pronounced interference with muscle contraction and its ready availability make it the most suitable of the two agents we tested for regional surgical anesthesia.

#### Euthanasia

Euthanasia protocols are often dictated by the need for unadulterated tissue harvest post-mortem. We recognize that in such cases, the use of a drug agent for initial anesthesia is undesirable. Likewise, we recognize that intact pieces of CNS tissue are a common requirement of cephalopod research. However, euthanasia that does not involve these restrictions can be humanely and rapidly achieved by immersion of the animal in isotonic magnesium chloride followed by the secondary method of surgical decerebration, as long as at least 5 min have elapsed from the cessation of respiration until the onset of the surgical method of killing. We caution that the animals on which we validated this procedure are small, and that a longer period be used for larger animals.

## Conclusions and further work needed

We have demonstrated that non-invasive monitoring of neural signal during anesthesia is a highly reliable indicator of anesthetic efficacy. We show conclusively that two commonly-used substances are effective as general, anesthetic agents in cephalopods, and two substances are effective as local anesthetics, but there is considerable work still needed to validate other sedating substances, which may or may not have anesthetic properties (clove oil, Seol et al., 2007; Gonçalves et al., 2012; and isoflourane, Polese et al., 2014, for example), and determine the full duration of local anesthetic effects. A major ongoing challenge for cephalopod research in nations where approval is required for procedures, is the use of long-lasting analgesics whose effects have been thoroughly tested. We expect that with the minimally-invasive and reversible neural recording procedures such as those described here, a range of candidate analgesics could be tested and approved relatively easily.

Magnesium chloride and ethanol are cheap, readily available, require no special equipment to use, and can be used by any experimenter without the need for extensive training. Given that cephalopods in research laboratories may not be under the direct care of veterinarians, obtaining prescription anesthesia drugs can be challenging for researchers. Thus, the evidence we supply here, that these readily available agents are effective at achieving immobility, loss of consciousness, and blockade of afferent sensory signal, represents a vitally important advance for the field of cephalopod research.

## Ethics statement

This study was conducted in the USA. It is exempt from US animal welfare regulations, as all animals used were invertebrates.

## Author contributions

RC: Designed study, conducted experiments, analyzed data, wrote the paper; HB-S, SB and NJ: Conducted experiments, analyzed data, wrote the paper; HB-S and SB: Contributed equally to this work.

### Conflict of interest statement

The authors declare that the research was conducted in the absence of any commercial or financial relationships that could be construed as a potential conflict of interest. The reviewer GP and handling Editor declared their shared affiliation.

## Supplementary material

Figure S1 Hook electrodes in place, showing position on the pallial nerve. **(A)** Ventral view of cuttlefish (post-mortem), with ventral mantle incised to expose the nerve. In experimental animals no incisions were made. **(B)** In octopuses the pallial nerve is rostral, large, and easily identified close to the mantle margin. This animal is alive and fully anesthetized in ethanol.

Figure S2 Still image sequence of an unrestrained cuttlefish undergoing progressive ethanol anesthesia and reversal. Timer shows the total time elapsed.

Figure S3 Still image sequence of an unrestrained cuttlefish undergoing magnesium chloride anesthesia and reversal. Timer shows the total time elapsed.

Figure S4 Still image sequence of an unrestrained octopus undergoing progressive ethanol anesthesia and reversal. Timer shows the total time elapsed.

Figure S5 Still image sequence of an unrestrained octopus undergoing magnesium chloride anesthesia and reversal. Timer shows the total time elapsed.

Figure S6 Electrophysiological traces from a cuttlefish undergoing chilled (4°C) seawater induction. There was an immediate burst of high-frequency firing in response to the cold water, which persisted for at least 5 min after immersion. This experiment was terminated at 5 min.
