# Supplementary figures and images for: In Vivo Recording of Neural and Behavioral Correlates of Anesthesia Induction, Reversal, and Euthanasia in Cephalopod Molluscs
Source: Front Physiol. 2018 Feb 20;9:109. doi: 10.3389/fphys.2018.00109 (PMC5826266; doi:10.3389/fphys.2018.00109)

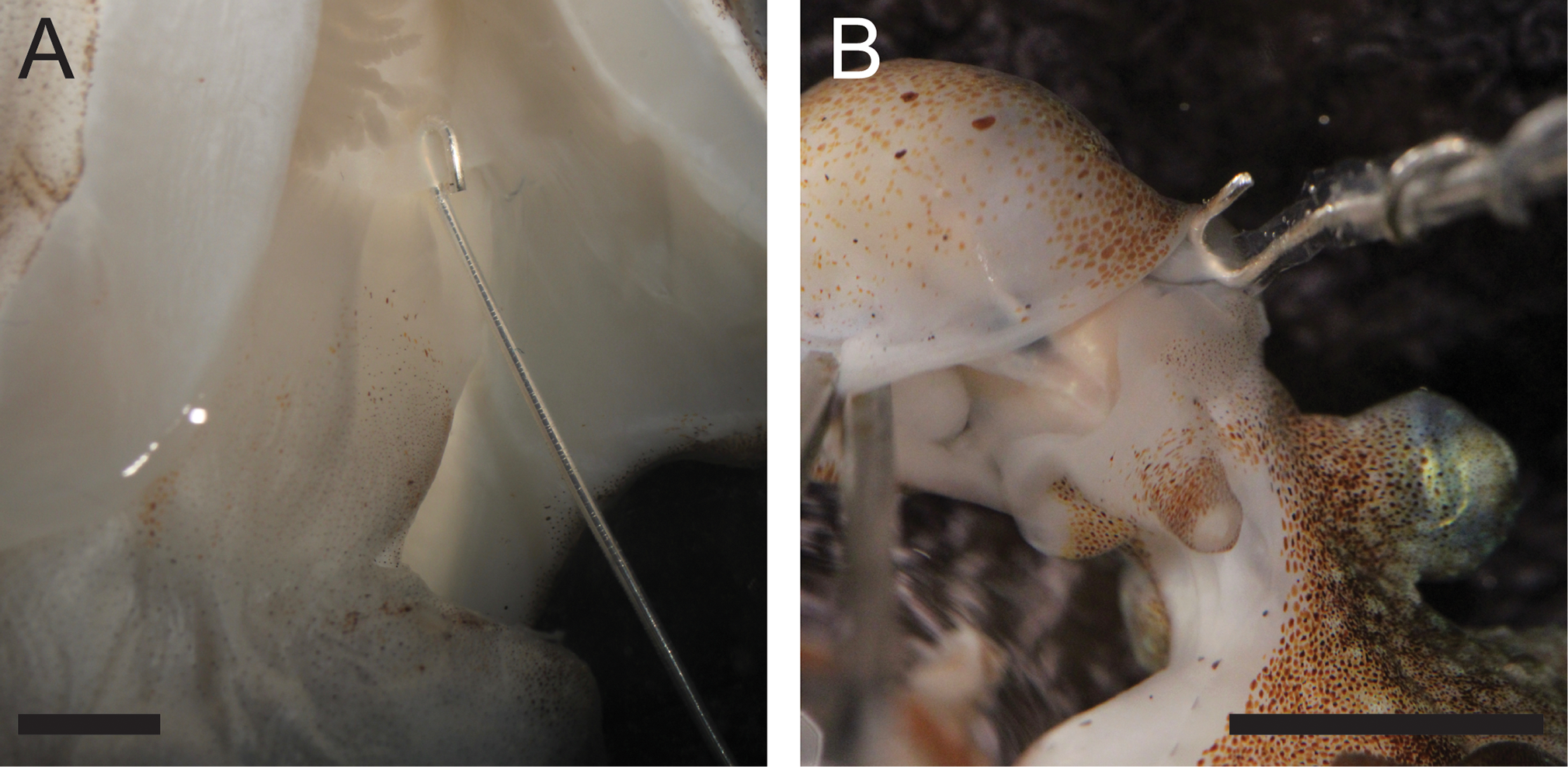

Supplement: Figure S1 — Hook electrodes in place, showing position on the pallial nerve. (A) Ventral view of cuttlefish (post-mortem), with ventral mantle incised to expose the nerve. In experimental animals no incisions were made. (B) In octopuses the pallial nerve is rostral, large, and easily identified close to the mantle margin. This animal is alive and fully anesthetized in ethanol. [file Image1.TIF]

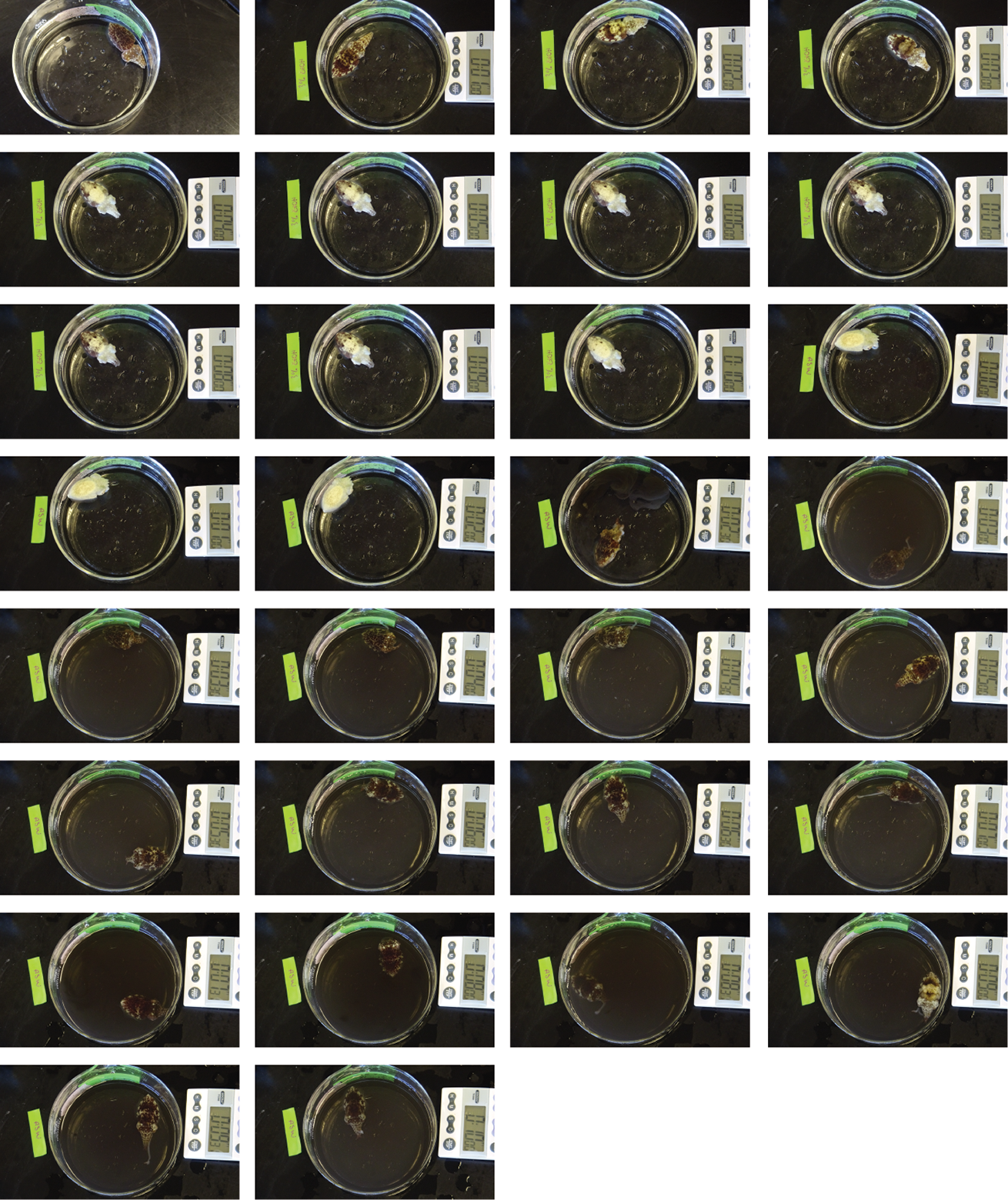

Supplement: Figure S2 — Still image sequence of an unrestrained cuttlefish undergoing progressive ethanol anesthesia and reversal. Timer shows the total time elapsed. [file Image2.TIF]

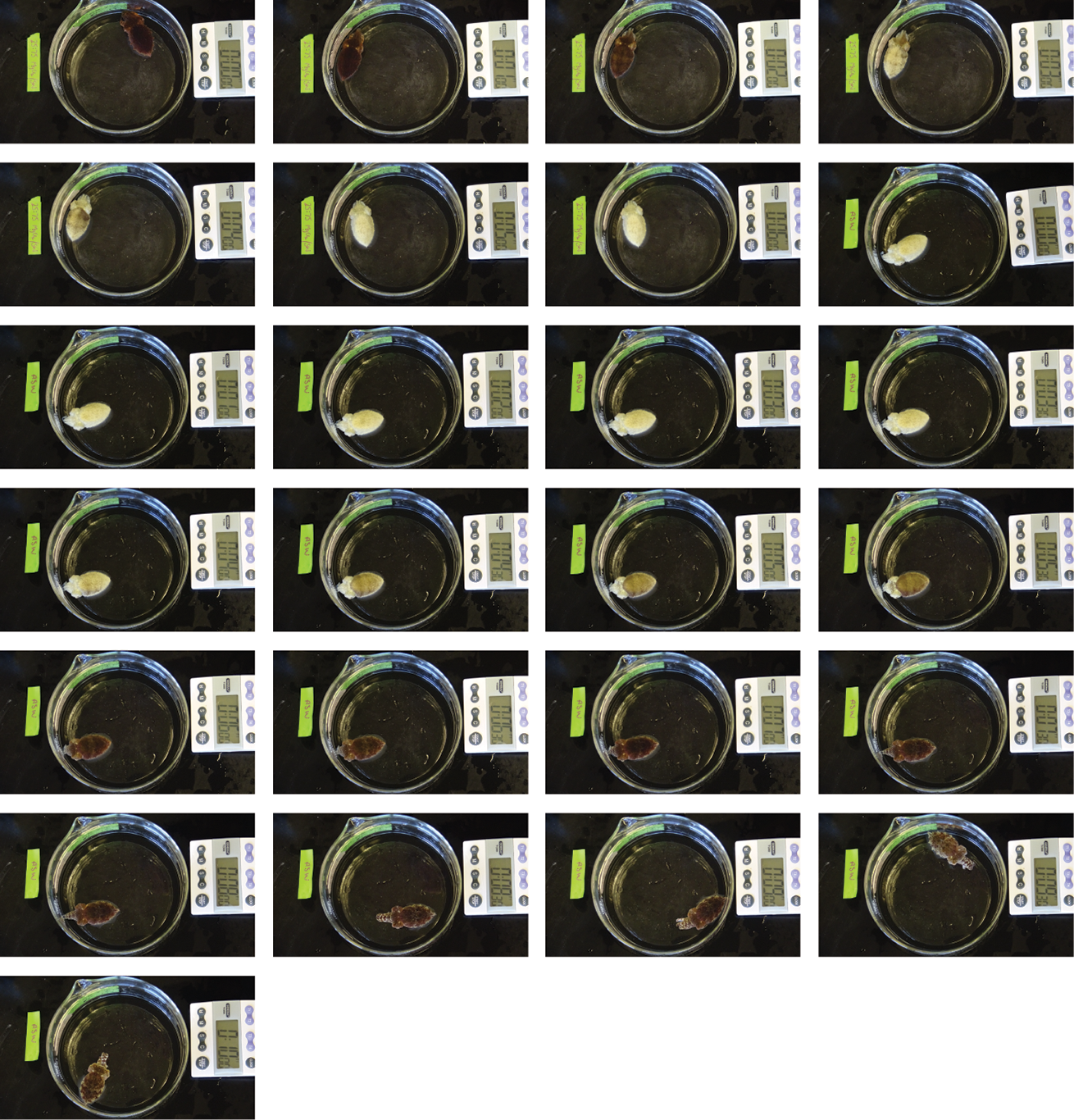

Supplement: Figure S3 — Still image sequence of an unrestrained cuttlefish undergoing magnesium chloride anesthesia and reversal. Timer shows the total time elapsed. [file Image3.TIF]

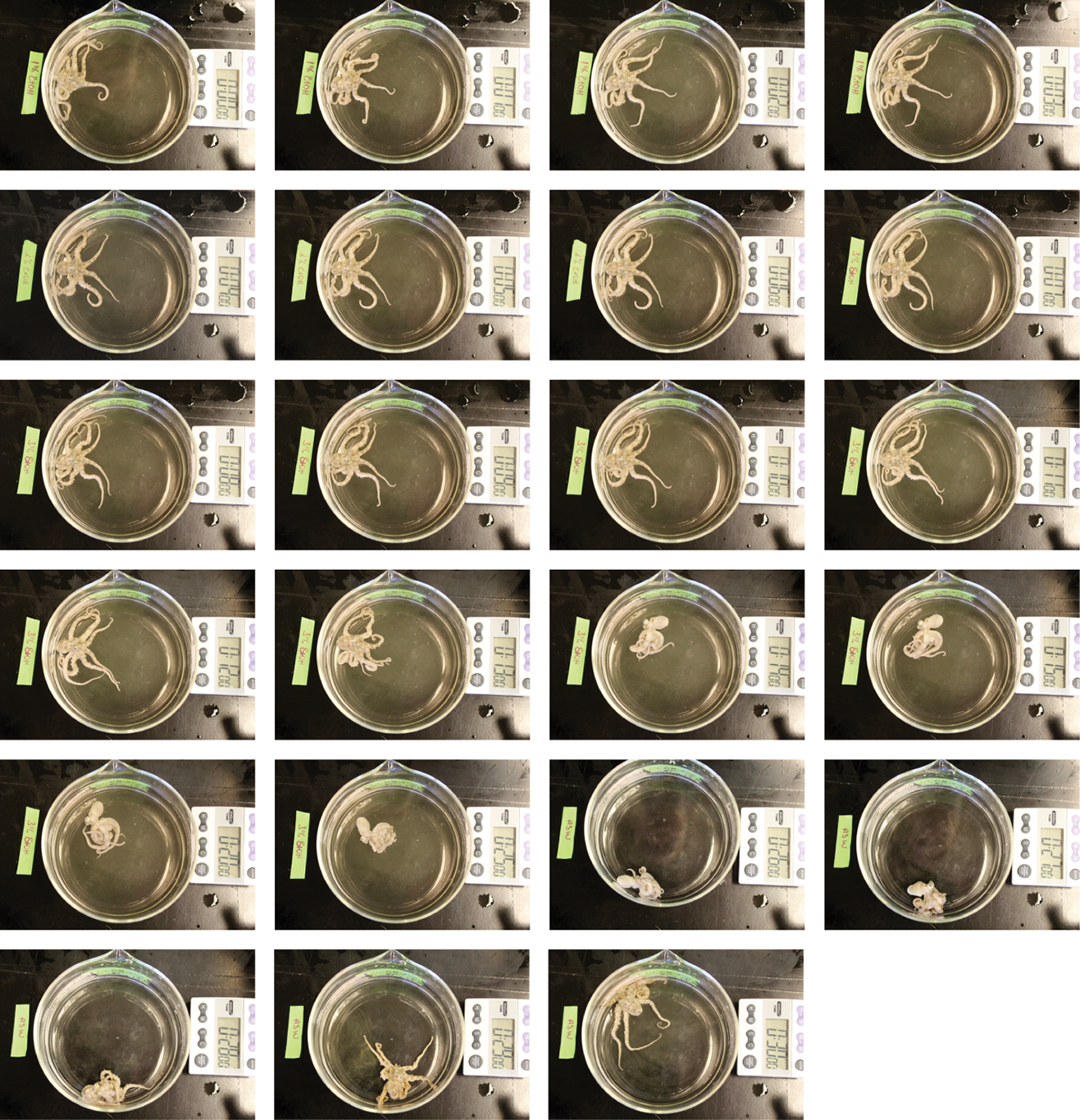

Supplement: Figure S4 — Still image sequence of an unrestrained octopus undergoing progressive ethanol anesthesia and reversal. Timer shows the total time elapsed. [file Image4.TIF]

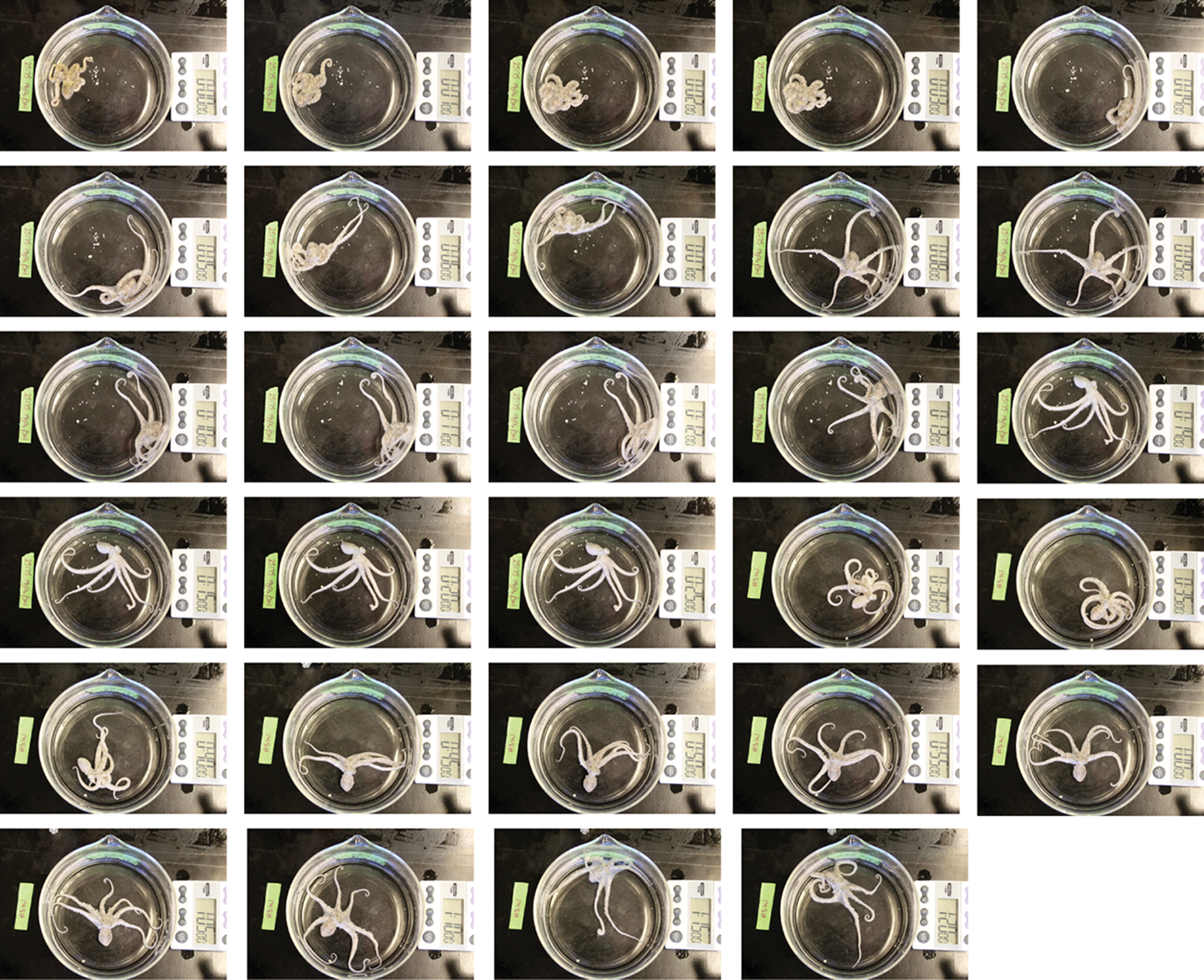

Supplement: Figure S5 — Still image sequence of an unrestrained octopus undergoing magnesium chloride anesthesia and reversal. Timer shows the total time elapsed. [file Image5.TIF]

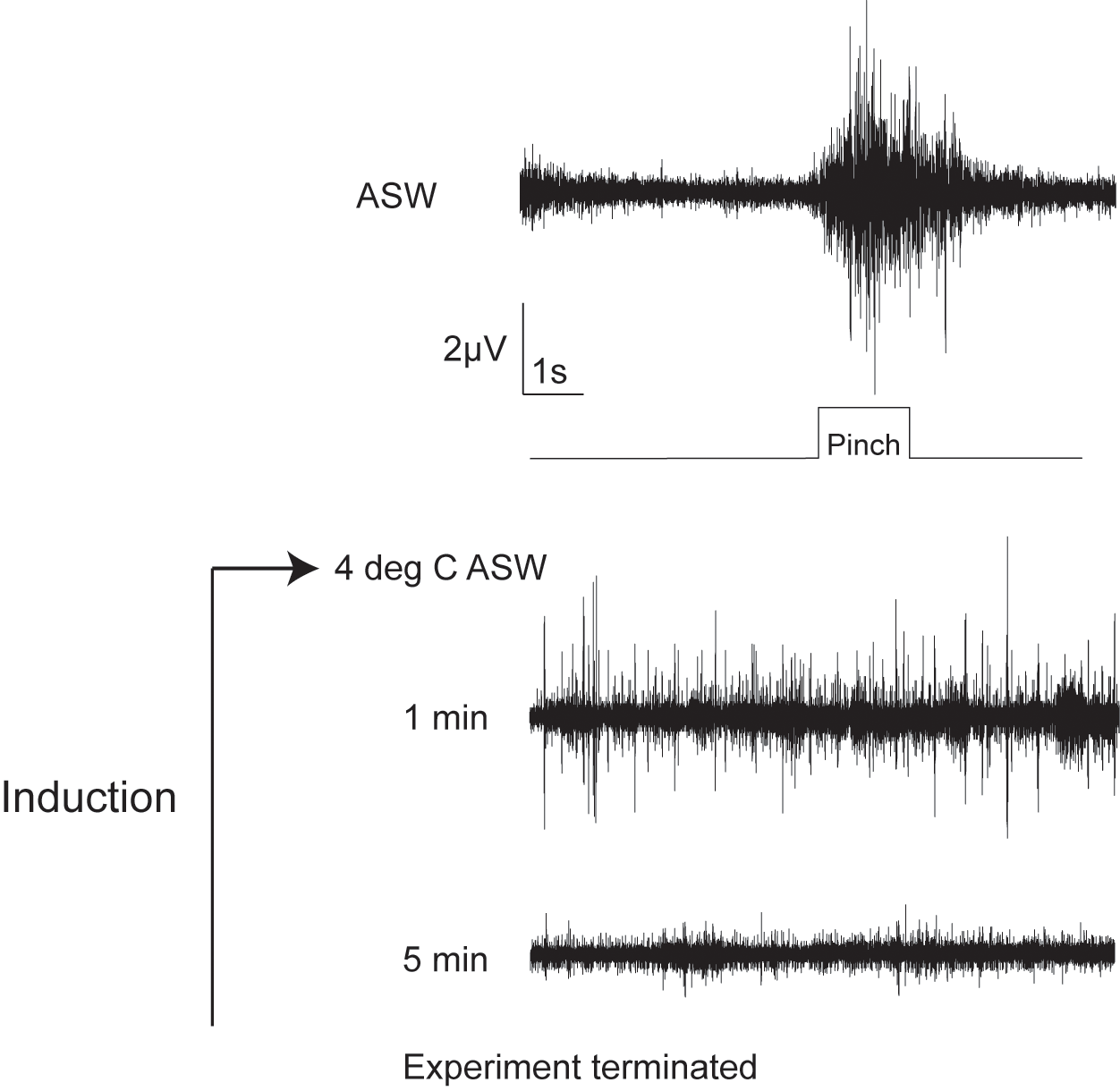

Supplement: Figure S6 — Electrophysiological traces from a cuttlefish undergoing chilled (4°C) seawater induction. There was an immediate burst of high-frequency firing in response to the cold water, which persisted for at least 5 min after immersion. This experiment was terminated at 5 min. [file Image6.TIF]
